# Prenatal Diagnosis of Mucopolysaccharidosis-Plus Syndrome (MPSPS)

**DOI:** 10.3390/genes14081581

**Published:** 2023-08-03

**Authors:** Viktoriia Sofronova, Lyutsiya Gotovtseva, Anastasia Danilova, Aitalina Sukhomyasova, Takahito Moriwaki, Seigo Terawaki, Takanobu Otomo, Nadezhda Maksimova

**Affiliations:** 1 Laboratory of Molecular Medicine and Human Genetics, North-Eastern Federal University, 677013 Yakutsk, Russia; 2 Department of Molecular and Genetic Medicine, Kawasaki Medical School, Kurashiki 701-0192, Japan; 3 Medical Genetics Center, Republic Hospital No. 1—National Center of Medicine, 677019 Yakutsk, Russia

**Keywords:** mucopolysaccharidosis, MPSPS, VPS33A, prenatal diagnosis, the Yakuts

## Abstract

Mucopolysaccharidosis-plus syndrome (MPSPS) is an autosomal-recessive disorder caused by c.1492C>T (p.R498W) in the *VPS33A* gene. MPSPS is a severe disorder that causes a short lifespan in patients. Currently, there is no specific treatment for patients. The Yakut population is more prone to this disease than others. Diagnosing MPSPS relies on clinical manifestations, and genetic testing (GT) is used to confirm the diagnosis. In this research, we examined two pregnancy cases, one of which involved a prenatal diagnosis for MPSPS. Notably, neither pregnant woman had a known family history of the disorder. During their pregnancies, both women underwent prenatal ultrasonography, which revealed increased prenasal thickness during the second trimester. In the first case, ultrasonography indicated increased prenasal thickness in the second trimester, but a definitive diagnosis was not made at that time. The patient was eventually diagnosed with MPSPS at 11 months of age. On the contrary, in the second case, GT uncovered that the parents were carriers of MPSPS. Consequently, a placental biopsy was performed, leading to an early diagnosis of MPSPS. This study emphasizes the importance of ultrasonography findings in prenatal MPSPS diagnosis. Combining ultrasonography with GT can be a valuable approach to confirming MPSPS at an early stage, allowing for the appropriate planning of delivery methods and medical care. Ultimately, this comprehensive approach can significantly enhance the quality of life of both affected patients and their parents.

## 1. Introduction

Mucopolysaccharidosis-plus syndrome (MPSPS; OMIM #617303) is a rare autosomal-recessive disorder characterized by the presence of clinical and biochemical features of mucopolysaccharidosis (MPS), including dysostosis multiplex, distinctive facial features, cardiopulmonary disorders, psychomotor retardation, increased levels of glycosaminoglycans (GAG) in the urine and plasma and additional “Plus” symptoms such as renal failure and hematopoietic abnormalities [1,2,3,4]. The first description of this disorder was reported by Gurinova et al., in 2014 [5]. The causative variant of the disease, c.1492C>T (p.R498W), in the vacuolar protein sorting-associated protein 33A (*VPS33A*) gene was identified in 2017 [1,2].

Children with MPSPS suffer from complications of the disorder and have a low life expectancy (10–20 months) due to the absence of specific treatments [6]. However, it was reported that a Moroccan patient with MPSPS [7] survived to his sixth year of age after treatment with a steroid and had significantly improved life expectancy compared to Yakut patients with MPSPS [6]. A total of 21 patients have been reported in the world, including 18 cases in Yakutia among non-consanguineous Yakut families [1,3,4,5,6], two cases in Turkey from a Turkish consanguineous family [2] and one case in Italy from a Moroccan consanguineous family [7]. The homozygous variant c.1492C>T (p.R498W) in the *VPS33A* gene was identified in all patients after birth. The diagnosis of MPSPS was based on clinical manifestation and the confirmation of a homozygous variant in the *VPS33A* gene.

Pavlova et al., have described the one possible variant, c.599G>C (p.R200P), in the *VPS33A* gene that might lead to MPSPS [8]. However, the patient revealed a mild phenotype and slow progress of the disease, which does not resemble MPSPS patients with variant c.1492C>T (p.R498W).

Yakuts show a high prevalence of some Mendelian disorders, including 3M syndrome (OMIM #273750), SOPH syndrome (OMIM #614800), methemoglobinemia type 1 (OMIM #250800), autosomal-recessive 1a deafness (OMIM #220290), myotonic dystrophy (OMIM #160900), oculopharyngeal muscular dystrophy (OMIM #164300) and spinocerebellar ataxia (OMIM #164400) [9,10,11,12,13,14]. The reason for their high prevalence in the Yakut population is the “founder effect”, which occurs due to high insulation [13,14].

The incidence rate of MPSPS in the Yakut population is predicted to be 1 in 12,000 births, and the frequency of allele c.1492C>T (p.R498W) in *VPS33A* was revealed to be relatively high (1:81) among the Yakut population [6]. Since 2017, there has been a genetic test (GT) for the confirmation of MPSPS in Yakutia. The possibility of other lysosomal storage disorders can be determined using enzyme activity tests or substrate level analysis [15,16]. MPSPS patients revealed unchanged lysosomal enzyme activity, but increased levels of GAG in their urine and plasma. However, biochemical tests or other possible analyses for the early detection of GAG in MPSPS-affected patients have not been established yet. 

The purpose of this report is to show the importance of the combination of prenatal ultrasonography (US) with GT to detect MPSPS in a given family without a history of MPSPS.

## 2. Results

### 2.1. Case Presentation 1

A 27-year-old pregnant Yakut woman underwent US in the second trimester where increased prenasal thickness was detected. The combined first trimester screening (CFTS) suggested a moderate risk (1:465) for Trisomy 21 (Table 1). Genetic counseling was offered due to the abnormal US findings and moderate risk for Trisomy 21.

**Figure 1:**
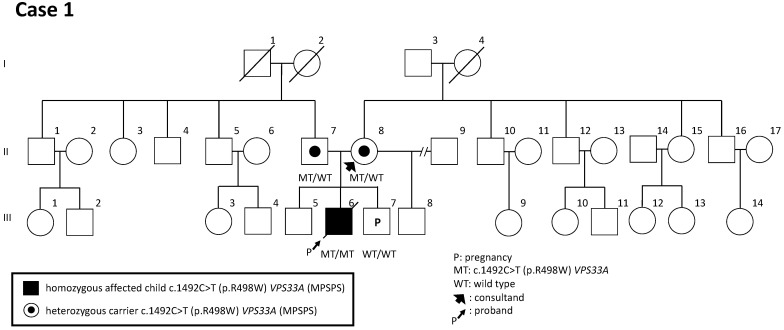
Family tree of case 1. The pedigree of case 1 presents the situation after confirmation of variant c.1492C>T (p.R498W) in *VPS33A* in proband (III-6). The next fetus (III-7) was not affected by the variant in *VPS33A*.

The family history was not burdened at that time (in 2016). Due to the US features and moderate risk for Trisomy 21, the woman underwent invasive prenatal diagnosis (IPD) via placenta biopsy to determine the karyotypic status of the fetus. The resulting karyotype was normal and showed 46,XY [11/11]. The baby was delivered at the 39th week of pregnancy. Starting from 2 months of age, the boy suffered from bronchitis. At the age of 11 months, MPSPS was suspected due to clinical manifestations such as stiffness of joints in the hands, distinctive facial features and hepatosplenomegaly. GT, which was available in 2017, confirmed a homozygous variant of c.1492C>T (p.R498W) in the *VPS33A* gene in the boy (III-6); then, a heterozygous variant was identified in both parents (II-7, II-8) (Figure 1). The boy died at the age of 18 months.

Six years later, the woman underwent chorionic biopsy and GT for her next pregnancy to determine the possibility of a *VPS33A* homozygous variant in her first trimester of pregnancy. Due to an already burdened family history, IPD was performed in the first trimester of pregnancy. Thus, the variant was not detected in both alleles, excluding MPSPS in the fetus (III-7) (Figure 1).

### 2.2. Case Presentation 2

**Figure 2:**
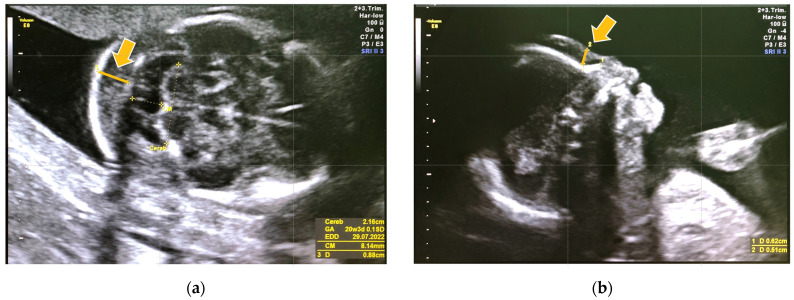
Prenatal ultrasonography of case 2 in the second trimester. (**a**) Nuchal fold thickness is the area of tissue at the back of the fetus (indicated by an arrow). (**b**) Prenasal thickness is the shortest distance between the anterior edge of the lowest part of the frontal bone and the facial skin anteriorly (indicated by an arrow). Both of them were increased.

A 23-year-old woman from a Yakut family underwent US in her second trimester (at 20 weeks and 5 days). CFTS showed a low risk for trisomy 21 (Table 1). US revealed an increased thickness of the nuchal fold and prenasal thickness (Figure 2). Genetic counseling was offered due to the abnormal US features.

**Figure 3:**
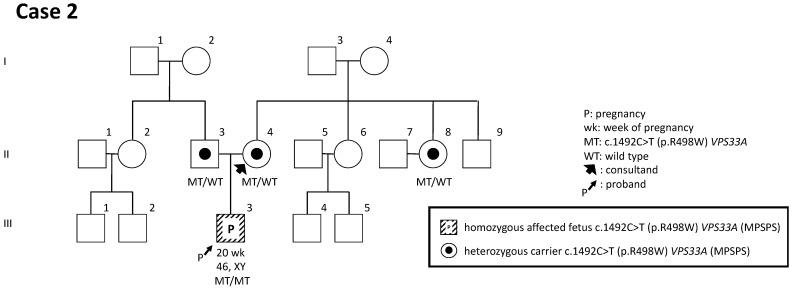
Family tree of case 2. The pedigree demonstrates the situation during pregnancy in which GT was performed for the parents, and the fetus was examined and identified as MPSPS affected the fetus in the second trimester of the pregnancy.

There was no family history of the inherited disorder. According to reports [20] stating, some inherited metabolic disorders have increased nuchal thickness in the first trimester, and doctors tested the parents to identify whether they were carriers of c.1492C>T (p.R498W). GT was performed on the woman and her husband to exclude the possibility of their carrying c.1492C>T in *VPS33A*. The test showed that both of them had a heterozygous variant of c.1492C>T in the *VPS33A* gene. Due to the autosomal-recessive inheritance of MPSPS and the possible 25% risk of it occurring in the next generation, IPD was performed. The karyotype was normal and showed 46,XY [15/15], and homozygous c.1492C>T p.R498W in the *VPS33A* gene was detected (III-3) (Figure 3).

The family learned about the course of MPSPS and decided to terminate the pregnancy according to the law and list of medical indications where pregnancy can be terminated regardless of the gestational age [21,22], with subsequent GT of fetal tissues.

## 3. Discussion

The Republic of Sakha (Yakutia) is located in the Far East of Russia, along the Arctic Ocean. The Yakuts are one of the most numerous indigenous ethnic groups in Siberia, and they make up the majority of the indigenous population of the Republic of Sakha (Yakutia). The Yakut population is a genetic insula, with its unique geographical location and specific history having led to a relatively higher prevalence of genetic disorders caused by specific pathogenic variants that are rarely found or cannot be detected in other populations [13,14,23]. MPSPS is one of the autosomal-recessive disorders with a high prevalence in the Yakut population. This disorder has severe manifestations that lead to death in early childhood. The pathophysiology of MPSPS is still unclear, and there is no specific therapy for MPSPS patients. Thus, only symptomatic treatment is available. In addition, MPSPS has no diagnostic method for its detection in a non-burdened family. Most carrier couples of disorders with recessive patterns of inheritance do not know their status due to an unburdened family history, and therefore, they are unaware of their reproductive risks. It is common for couples to find out about their carrier status only after the birth of an affected child [24]. It is speculated that the early diagnosis of MPSPS can lead to early prevention of the complications of MPSPS and extend the lifespans of patients. There is a report describing one Moroccan child who was the eldest MPSPS patient and whose psychomotor milestones and quality of life were improved by prolonged steroid treatment [7]. However, this single reported case cannot conclude that there is a possible treatment for all MPSPS patients. On the other hand, according to the reported Moroccan case, we might have a chance to prevent the appearance of neurological symptoms in the early stage of life and improve quality of life via early observations in already-diagnosed children before MPSPS manifestations. The ultrasonographic examination of fetuses and placentas plays a major role in the diagnosis of metabolic disorders. There are several prenatal cases of Niemann–Pick type C (NPC; OMIM #257220) where fetal ascites and hepatosplenomegaly were detected in the second trimester of pregnancy [25] and cystic hygroma in the first trimester [26]. Among mucopolysaccharidosis (MPS), MPS VII (Sly syndrome; OMIM #253220) revealed nonimmune hydrops fetalis [27]. Our MPSPS cases did not display these specific abnormalities, underscoring the need for a combined approach of prenatal US with GT for early detection.

In case 1, GT was unavailable due to the unidentified causative variant for MPSPS at that time. As a result, MPSPS remained undetected despite some abnormal US features in the fetus. However, in case 2, abnormal US features were detected during the second trimester, highlighting the significance of identifying such features to prompt suspicion of MPSPS. Increased prenasal thickness and thickness of the nuchal fold may serve as potential US markers requiring further investigation for MPSPS during the second trimester. However, how many MPSPS patients show these specific US findings is yet to be determined due to limited prenatal data.

GT does have some limitations that need to be addressed. Currently, it can only identify a single variant, c.1492C>T (p.R498W), in the *VPS33A* gene. Whole-exome sequencing could be used for parents and prenatal testing, but its cost is prohibitive of widespread use. Additionally, in the Republic of Sakha, GT was only available for pregnant women who received genetic counseling and participated in the Genetic Carrier Screening (GCS) pilot program in specific districts of Yakutia [28]. Expanding the use of GT or GCS during pregnancy would allow families to be aware of their reproductive risks and make informed decisions.

One advantage of GT is the ability to identify carriers among relatives and siblings within families. For example, a sibling (II-8) was determined to be a carrier before her pregnancy (Figure 3). When both parents are found to have pathogenic variants before conception, they have several options to consider, including avoiding pregnancy, using gametes from a noncarrier donor, employing preimplantation genetic diagnosis to transfer only unaffected embryos, or undergoing prenatal diagnosis while conceiving naturally if desired.

While there are currently no prenatal therapies to alleviate the symptoms of MPSPS, GT can provide valuable information to families, reassuring them when their baby does not have the genetic disease. In fact, in the case of the MPSPS-affected family (case 1), they underwent IPD in the first trimester during their subsequent pregnancy. For families with MPSPS burden, informed decisions can be made during subsequent pregnancies, leading to better care and management of affected individuals, and potentially improving their outcomes and quality of life, as seen in the Moroccan patient’s case [7].

In conclusion, the presented clinical cases underscore the importance of the early detection of MPSPS through a combined approach of prenatal US and GT, particularly in families without a history of the disorder. This approach has the potential to enhance medical care and management, resulting in improved outcomes and quality of life for affected individuals.

## 4. Materials and Methods

### 4.1. Patients and Medical Cards

A retrospective review of medical cards (case 1 from 2016, case 2 from 2022) was performed. US was conducted using a Voluson™ E8 Expert (“GE Healthcare Austria GmbH & Co OG”, Wien, Austria) with a Wide Band Convex Ultra-Light Volume Transducer RAB6-D 2–7 MHz. Peripheral blood samples of the parents were obtained for molecular GT, and placental samples were collected for molecular GT and karyotyping.

### 4.2. Molecular Genetic Test

This study was conducted with the voluntary informed consent of all individuals. Sampling of peripheral blood (9 mL) was carried out in a “VACUETTE” vacuum tube containing K3-EDTA as an anticoagulant (“Greiner Bio-One”, Kremsmünster, Austria). Extraction of DNA from peripheral blood and placental sample was performed with two methods using commercial “ExtraDNA” kits (“Evrogen”, Moscow, Russia) and the method of phenol–chloroform extraction, respectively [29].

GT for c.1492C>T (p.R498W) was performed using real-time PCR on a CFX96 Touch Real-Time PCR Detection System (“Bio-Rad”, Hercules, California, USA). Commercial kits of reagents for DNA amplification were applied (“TestGen”, Ulyanovsk, Russia). PCR conditions: first, denaturation took place at 95 °C (2 min), for 40 amplification cycles: denaturation temperature was 95 °C (10 s), and annealing and extension temperature was 60 °C (20 s). A specific primer set (forward: 5’-CCAAAAGGCCACAGTCAGGT-3’, reverse: 5’-TCTGTAGATGCTGGACTGGGA-3’) and specific hydrolysis probes (wild type (c.1492C): 5’- ROX-CTCAGTGTGCGGCTGGCCCA-BHQ2-3’, mutant (c.1492T): 5’-FAM-CTCAGTGTGTGGCTGGCCCA-RTQ1-3’) were used.

## Figures and Tables

**Table 1:** Comparative table of two cases of pregnancy.

Parameters	Case 1	Case 2
Year	2016	2022
Ethnicity	Yakut	Yakut
Age of the woman	27	23
Family history	Non-burdened for MPSPS	Non-burdened for MPSPS
MPSPS diagnosis using GT	Child at the age of 11 months	Fetus at the age of 20 weeks
Trimester I		
CRL	51 mm	52.2 mm
NT	1.5 mm (95th P = 2.32 mm) [17]	1.8 mm (95th P = 2.32 mm) [17]
NB	Detectable	Detectable
beta-hCG	2.2 MoM (0.5–2.0 MoM)	1.7 MoM (0.5–2.0 MoM)
PAPP-A	0.4 MoM (0.5–2.0 MoM)	0.7 MoM (0.5–2.0 MoM)
Risk for Trisomy 21	1:465	1:19,000
Trimester II		
US features		
Prenasal thickness	Increased	5.1 mm (95th P = 3.3 mm) [18]
Nasal bone length	In reference range	6.2 mm (5th P = 5.6 mm) [18]
Nuchal fold thickness	In reference range	8.8 mm (< 6 mm) [19]
GT for partners	NA	Heterozygous c.1492C>T (p.R498W) in *VPS33A* in both partners
Placental biopsy		
Karyotyping	46,XY [11/11]	46,XY [15/15]
GT	NA	Homozygous c.1492C>T (p.R498W) in *VPS33A*

MPSPS, mucopolysaccharidosis-plus syndrome; GT, genetic test; CRL, crown–rump length; NT, nuchal translucency; NB, nasal bone; beta-hCG, beta human chorionic gonadotrophin; PAPP-A, pregnancy-associated plasma protein-A; US, ultrasonography; NA, not available; P, percentile.

## Data Availability

All relevant data that support the findings of this study are within the manuscript.

## Abbreviation

beta-hCG	Beta-human chorionic gonadotrophin
CFTS	Combined first trimester screening
CRL	Crown–rump length
GAG	Glycosaminoglycan
GT	Genetic test
GCS	Genetic carrier screening
IPD	Invasive prenatal diagnosis
MPSPS	Mucopolysaccharidosis-plus syndrome
NT	Nuchal translucency
NB	Nasal bone
PAPP-A	Pregnancy-associated plasma protein A
US	Ultrasonography
VPS33A	Vacuolar protein sorting-associated protein 33A
